# Continuous renal replacement therapy *versus* intermittent hemodialysis as first modality for renal replacement therapy in severe acute kidney injury: a secondary analysis of AKIKI and IDEAL-ICU studies

**DOI:** 10.1186/s13054-022-03955-9

**Published:** 2022-04-04

**Authors:** Stéphane Gaudry, François Grolleau, Saber Barbar, Laurent Martin-Lefevre, Bertrand Pons, Éric Boulet, Alexandre Boyer, Guillaume Chevrel, Florent Montini, Julien Bohe, Julio Badie, Jean-Philippe Rigaud, Christophe Vinsonneau, Raphaël Porcher, Jean-Pierre Quenot, Didier Dreyfuss

**Affiliations:** 1 Département de Réanimation Médico-Chirurgicale, APHP Hôpital Avicenne, Bobigny, France; 2 Health Care Simulation Center, UFR SMBH, Université Sorbonne Paris Nord, Bobigny, France; 3 Present Address: Common and Rare Kidney Diseases, INSERM, UMR-S 1155, Hôpital Tenon, Sorbonne Université, 4 rue de la Chine, 75020 Paris, France; 4 Investigation Network Initiative–Cardiovascular and Renal Clinical Trialists, Bobigny, France; 5 Centre of Research in Epidemiology and Statistics (CRESS), Université de Paris, French Institute of Health and Medical Research (INSERM), National Institute of Agricultural Research (INRA), Paris, France; 6 Hôpital Caremeau, Nimes, France; 7 Réanimation Polyvalente, CHR départementale La Roche Sur Yon, La Roche sur Yon, France; 8 CHU Pointe-À-Pitre/Abymes, Pointe-a-Pitre, France; 9 Réanimation Et USC, GH Carnelle Portes de L’Oise, 95260 Beaumont sur Oise, France; 10 CHU de Bordeaux, Service de Réanimation Médicale, 33000 Bordeaux, France; 11 Réanimation Polyvalente, CH Sud Francilien, Corbeil Essones, France; 12 Réanimation Polyvalente, Centre Hospitalier d’Avignon, Avignon, France; 13 Anesthésie Réanimation Médicale et Chirurgicale, CH Lyon Sud, Pierre Benite, France; 14 Réanimation Polyvalente, Hôpital Nord Franche-Comte CH Belfort, Belfort, France; 15 Réanimation Polyvalente, CH de Dieppe, Dieppe, France; 16 Médecine Intensive Réanimation, CH Bethune Beuvry – Germont et Gauthier, Bethune, France; 17 Department of Intensive Care, François Mitterrand University Hospital, Dijon, France; 18 Lipness Team, INSERM Research Center LNC-UMR1231 and LabExLipSTIC, University of Burgundy, Dijon, France; 19 INSERM CIC 1432, Clinical Epidemiology, University of Burgundy, Dijon, France; 20 Médecine Intensive-Réanimation, APHP, Hôpital Louis Mourier, Université de Paris, Colombes, France

**Keywords:** Renal replacement therapy, Acute kidney injury, Critical care

## Abstract

**Background:**

Intermittent hemodialysis (IHD) and continuous renal replacement therapy (CRRT) are the two main RRT modalities in patients with severe acute kidney injury (AKI). Meta-analyses conducted more than 10 years ago did not show survival difference between these two modalities. As the quality of RRT delivery has improved since then, we aimed to reassess whether the choice of IHD or CRRT as first modality affects survival of patients with severe AKI.

**Methods:**

This is a secondary analysis of two multicenter randomized controlled trials (AKIKI and IDEAL-ICU) that compared an early RRT initiation strategy with a delayed one. We included patients allocated to the early strategy in order to emulate a trial where patients would have been randomized to receive either IHD or CRRT within twelve hours after the documentation of severe AKI. We determined each patient’s modality group as the first RRT modality they received. The primary outcome was 60-day overall survival. We used two propensity score methods to balance the differences in baseline characteristics between groups and the primary analysis relied on inverse probability of treatment weighting.

**Results:**

A total of 543 patients were included. Continuous RRT was the first modality in 269 patients and IHD in 274. Patients receiving CRRT had higher cardiovascular and total-SOFA scores. Inverse probability weighting allowed to adequately balance groups on all predefined confounders. The weighted Kaplan–Meier death rate at day 60 was 54·4% in the CRRT group and 46·5% in the IHD group (weighted HR 1·26, 95% CI 1·01–1·60). In a complementary analysis of less severely ill patients (SOFA score: 3–10), receiving IHD was associated with better day 60 survival compared to CRRT (weighted HR 1.82, 95% CI 1·01–3·28; *p* < 0.01). We found no evidence of a survival difference between the two RRT modalities in more severe patients.

**Conclusion:**

Compared to IHD, CRRT as first modality seemed to convey no benefit in terms of survival or of kidney recovery and might even have been associated with less favorable outcome in patients with lesser severity of disease. A prospective randomized non-inferiority trial should be implemented to solve the persistent conundrum of the optimal RRT technique.

**Supplementary Information:**

The online version contains supplementary material available at 10.1186/s13054-022-03955-9.

## Background

Acute kidney injury (AKI) complicates the course of many critically ill patients and is associated with both increased morbidity and mortality [1, 2]. The treatment of AKI is based on both conservative measures and timely use of renal replacement therapy (RRT). Intermittent hemodialysis (IHD) and continuous RRT (CRRT) with hemofiltration are the two main modalities in the intensive care unit (ICU). Controversy exists as to which is the optimal one in this setting [3–5]. Indeed, CRRT allows for slower fluid removal which may ensure better hemodynamic stability and slower control of solute concentration than IHD (thereby minimizing fluid shift). This is the reason why many critical care physicians favor CRRT, at least at the initial stage of ICU stay [6].

However, the COVID crisis has highlighted the shortage of RRT devices [7]. To overcome this problem, some authorities have suggested the use of CRRT for short periods (i.e., 12 h a day) in order to increase machine availability and the use of the same machine in two different patients in the same day [8]. A simpler alternative would be to favor IHD as it allows treating more than two patients (usually three) with the same machine in one day. Indeed, IHD may be well tolerated owing to simple interventions including isovolemic initiation, reduced dialysate temperature, preferential use of bicarbonate buffer, sodium profiling (i.e., dialysate [Na+] > 145 mmol/L) and conservative initial ultrafiltration [9].

Improvement in critical care delivery resulted in marked improvement of patient prognosis in recent years [10, 11]. For instance, mortality was substantially higher in both groups of a large RCT dating back 15 years that compared IHD and CRRT [12] than in recent RCTs on RRT timing where both modalities were used [13–15]. This justifies the reassessment of the impact of RRT modality on survival. New data can therefore be generated which will in turn generate new research questions and restimulate research interest in this area.

Our teams recently conducted two independent large multicenter RCTs on the timing of RRT initiation (AKIKI [13] and IDEAL-ICU [15] trials). The choice of RRT technique was left at clinician discretion at each study site, in accordance with French guidelines [16]. The present study merged the two datasets in order to evaluate whether the choice of IHD or CRRT as first modality affects survival of critically ill patients with severe AKI.

## Methods

### Study design and patients

This study is a secondary analysis of two open pragmatic multicenter RCTs (the AKIKI [13] and IDEAL-ICU [15] trials) that compared an early RRT initiation strategy with a delayed one. Both were multicenter studies involving critically ill patients with severe AKI among other organ failures.

Patients allocated to the early strategy received RRT less than 12 h after documentation of severe AKI. This allows for studying this patient population at a time when baseline characteristics are unlikely to have changed noticeably, resulting in measurable confounding we can account for.

Following from the principles of an ideal target RCT, we emulated a trial where patients would have been randomized to receive either IHD or CRRT within twelve hours after the documentation of severe AKI. In a bid to mimic the intention to treat analysis from a randomized trial, we determined each patient’s modality group as the first RRT modality they received. We then used propensity score-based analyses to correct for confounding.

The AKIKI trial and the IDEAL-ICU trial received approval for all participating centers from competent French legal authority (Comité de Protection des Personnes d’Ile de France VI, ID RCB 2013-A00765-40, NCT01932190 for AKIKI and Comité de Protection des Personnes Est I ID RCB 2012-A00519-34 for IDEAL-ICU), and consent of patient or relatives was obtained before inclusion (except in emergencies where deferred consent was allowed by the Institutional Review Board).

### Interventions

In both trials, the choice of RRT modality was left at clinician discretion and a switch to either modality was allowed over the course of a patient ICU stay. No patient was treated with hybrid machine such as sustained low efficiency dialysis (SLED). In the present study, we analyzed outcomes according to the first modality used. Investigators were encouraged to follow current guidelines [16–18]. All study centers used dialyzers with biocompatible membranes for IHD and CRRT. The recommended buffer in dialysate and replacement fluid was bicarbonate. Recommendations included the delivery of a Kt/V of 3·9 per week during IHD and an effluent volume of 20–25 mL/kg/h for CRRT.

### Outcomes

The primary outcome was 60-day survival measured from the date of initiation of RRT until death or day 60. Secondary outcomes were the status at ICU and hospital discharge, kidney recovery (defined as RRT discontinuation and spontaneous urine output higher than 1000 mL per 24 h in the absence of diuretic therapy or higher than 2000 mL per 24 h with diuretics) before day 28, survival with no need for RRT (RRT dependence) at day 28, length of stay in ICU, number of days free of RRT, mechanical ventilation and vasopressor at 28 days.

### Statistical analyses

The main analysis relied on inverse probability of treatment weighting (IPTW) [19, 20] to balance the differences in baseline characteristics between treatment groups. A propensity score model was estimated using logistic regression, with the first RRT modality as dependent variable and the following pre-randomization characteristics as covariates: age, gender, weight, comorbidities (heart failure, hypertension, diabetes, cirrhosis, respiratory disease, cancer, AIDS, organ graft), treatments with immunosuppressive drugs, severity (respiratory SOFA, cardiovascular SOFA, bilirubin SOFA, platelet SOFA, Glasgow SOFA, renal SOFA, global SOFA), urea plasma concentration, pH, creatinine plasma concentration before ICU admission, and trial (AKIKI or IDEAL-ICU). These variables were specified before outcome analyses. Polynomials were used to handle potentially nonlinear effects of continuous variables. Nonlinear terms were removed if they did not improve balance of groups after weighting. Standardized mean differences (SMD) were examined to assess balance between groups before and after weighting [21, 22], and a value below 10% was considered as indicating clinically meaningful balance of a covariate [23]. Causal treatment effects were assessed for the whole weighted population (i.e. average treatment effect) in terms of hazard ratio (HR), risk ratio (RR), absolute risk difference (ARD) or mean difference (MD). We estimated survival through a weighted Kaplan–Meier estimator and used restricted mean survival time (RMST) to measure the average survival time for each group over the 60 days follow-up period [24].

One difficulty stemmed from the fact that some centers only provided one of the two RRT modalities, possibly violating the positivity assumption, which is crucial for propensity score-based methods. We, therefore, performed a sensitivity analysis estimating the treatment effects by overlap weighting (OW) [25] through a second propensity score model which accounted for centers. Specifically, we used a mixed-effects logistic regression model with the same aforementioned covariates as fixed effects and a random center effect.

The residual confounding effect that would be due to centers in the IPTW analysis was investigated through additional prognostic modelling and showed that very little confounding due to centers was expected (Additional file 1: Table S1 and Figure S2). The positivity assumption and between-group balance were further evaluated for each weighting technique by plotting the distribution of the propensity score in both groups (Additional file 1: Figures S3 and S4). Ninety-five percent confidence intervals (95% CI) were estimated by bootstrapping. Missing data were handled through multiple imputations by chained equations using outcomes as well as all confounders mentioned above in the imputation model. Because roughly 5% of patients had one or more missing predictors (Additional file 1: Figure S5), 5 independent imputed data sets were generated and analyzed separately. Estimates were then pooled using Rubin’s rules.

We assessed treatment effect heterogeneity, using the same methodology stratified by thirds of baseline risk as evaluated by SOFA scores.

All analyses were performed using the R statistical software version 4.0.0 or later (R Foundation for Statistical Computing, https://www.R-project.org/).

## Results

### Patients

A total of 543 critically ill patients with severe AKI who received RRT in the early strategy of our two previous studies were included in the present one (304 from the AKIKI trial and 239 from the IDEAL-ICU trial). The median time between random allocation to the early strategy and RRT initiation was 2 h (IQR, 1 to 3) and 3 h (IQR, 2 to 4) in AKIKI and IDEAL-ICU, respectively.

**Fig. 1:**
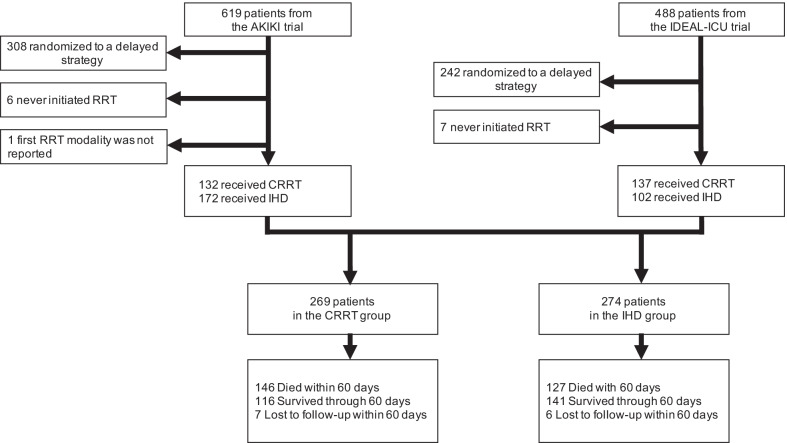
Study flowchart

Two hundred sixty-nine patients received CRRT and 274 IHD as first RRT modality (Fig. 1). Patient characteristics at baseline are depicted in Table 1. Patients receiving IHD were more frequently hypertensive, had higher serum creatinine levels and higher coagulation-SOFA scores at baseline. On the opposite, patients receiving CRRT had higher cardiovascular and total-SOFA scores.

**Table 1:** Patient characteristics at baseline. All characteristics reported in the table were determined at inclusion in the AKIKI or IDEAL-ICU trial, or before initiation of renal replacement therapy*

Characteristic	Before weighting				After inverse probability weighting			
	Total (*n* = 543)	CRRT group (*n* = 269)	IHD group (*n* = 274)	SMD (%)	CRRT group (*n* = 268.2)	IHD group (*n* = 271.9)	SMD (%)	*p* value
Age, years	66.5 (13.3)	66.2 (13.4)	66.8 (13.3)	4.4	66.5 (13.4)	66.7 (13.2)	1.5	0.87
Female sex	200 (36.8%)	101 (37.6%)	99 (36.1%)	2.9	97.4 (36.3%)	101.2 (37.2%)	1.9	0.83
Serum creatinine before ICU admission, µmol/L*	86.2 (34.1)	84·6 (36.7)	87.8 (31.3)	9.4	86.6 (33.8)	86.8 (32.1)	0.6	0.95
Coexisting conditions								
Chronic kidney disease	54 (9.9%)	23 (8.6%)	31 (11.3%)	9.3	24.3 (9.1%)	30.6 (11.3%)	7.2	0.43
Chronic hypertension	301 (55.4%)	136 (50.6%)	165 (60.2%)	19.5	148.4 (55.4%)	153.3 (56.4%)	2.0	0.82
Diabetes mellitus	109 (20.1%)	55 (20.4%)	54 (19.7%)	1.8	53.7 (20.0%)	53.5 (19.7%)	0.9	0.92
Congestive heart failure	43 (7.9%)	22 (8.2%)	21 (7.7%)	1.9	21.6 (8.1%)	22.2 (8.2%)	0.4	0.97
Cirrhosis	53 (9.8%)	27 (10.0%)	26 (9.5%)	1.8	26.3 (9.8%)	25.1 (9.2%)	2.0	0.82
Respiratory disease	62 (11.4%)	30 (11.2%)	32 (11.7%)	1.7	30.4 (11.3%)	31.3 (11.5%)	0.5	0.95
Cancer	89 (16.4%)	48 (17.8%)	41 (15.0%)	7.8	43.2 (16.1%)	43.2 (15.9%)	0.6	0.95
AIDS	5 (0.9%)	3 (1.1%)	2 (0.7%)	4.0	3.1 (1.1%)	4.0 (1.5%)	2.8	0.79
Immunosuppressive drugs	32 (5.9%)	21 (7.8%)	11 (4.0%)	16.1	15.3 (5.7%)	14.0 (5.2%)	2.4	0.79
Organ transplantation	5 (0.9%)	4 (1.5%)	1 (0.4%)	11.7	2.6 (1.0%)	2.6 (1.0%)	< 0.1	0.99
SOFA score at inclusion (0 to 24)	11.4 (3.2)	11.7 (3.1)	11.1 (3.3)	18.6	11.5 (3.2)	11.4 (3.2)	1.8	0.84
Renal SOFA (1 to 5)	3.7 (1.1)	3.6 (1.1)	2.9 (1.2)	4.4	3.7 (1.1)	3.7 (1.1)	0.4	0.97
Cardiovascular SOFA (1 to 5)	4.4 (1.3)	4.6 (1.1)	4.3 (1.4)	24.6	4.5 (1.2)	4.4 (1.3)	2.5	0.78
Liver SOFA (1 to 5)	1.8 (1.1)	1.8 (1.1)	1.8 (1.1)	4.0	1.8 (1.1)	1.8 (1.1)	3.1	0.73
Neurologic SOFA (1 to 5)	2.2 (1.5)	2.2 (1.5)	2.3 (1.5)	6.8	2.2 (1.5)	2.2 (1.5)	0.4	0.96
Coagulation SOFA (1 to 5)	3.2 (1.6)	3.0 (1.6)	3.3 (1.6)	17.3	3.2 (1.6)	3.1 (1.6)	2.1	0.81
Body weight, kg	82.0 (21.9)	81.1 (21.9)	83.0 (21.9)	8.8	83.0 (23.5)	82.3 (21.9)	3.3	0.73
Laboratory values								
Serum creatinine, µmol/L	286.6 (126.9)	273.7 (122.5)	299.3 (130.0)	20.3	287.5 (129.1)	290.2 (124.0)	2.1	0.82
Serum urea, mmol/L	19.8 (9.1)	19.4 (9.0)	20.3 (9.2)	10.1	19.8 (9.0)	20.0 (9.0)	1.5	0.87
Serum potassium, mmol/L	4.38 (0.77)	4.41 (0.78)	4.35 (0.76)	8.3	4.38 (0.77)	4.38 (0.79)	0.5	0.96
Arterial blood pH	7.30 (0.10)	7.30 (0.10)	7.30 (0.09)	4.3	7.30 (0.10)	7.30 (0.10)	1.3	0.89

Data are mean (SD) or *n* (%). *SMD* standardized mean difference, expressed as a percentage; *SOFA score* Sequential Organ Failure Assessment score

*The serum creatinine concentration before ICU admission was either determined with the use of values measured in the 12 months preceding the ICU stay or was estimated using the Modification of Diet in Renal Disease Study Group formula

Of the 269 patients who initially received CRRT, 56 (20.8%) switched to IHD on average at day 6.4. Of the 274 patients who initially received IHD, 48 (17.5%) switched to CRRT on average at day 5.7.

### Unweighted analyses

**Fig. 2:**
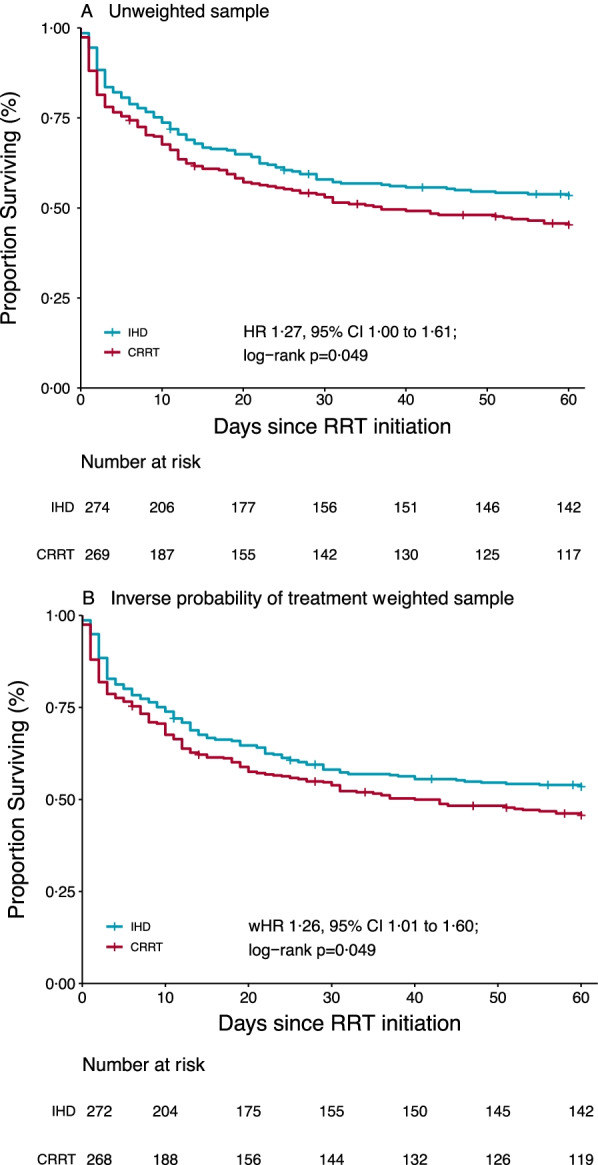
Primary outcome: probability of survival in the unweighted sample (**A**) and in the IPTW sample (**B**). *HR* hazard ratio, *IHD* intermittent hemodialysis, *CRRT* continuous renal replacement therapy

Through the 60-day follow-up period, 273 patients (50%) died (146 in the CRRT group and 127 in the IHD group). Thirteen patients (2%) were lost to follow-up. The Kaplan–Meier death rate at day 60 was 54.7% in the CRRT group and 46.5% in the IHD group (Fig. 2A) (HR 1.27, 95% CI 1.00 to 1.61) The ARD was 8.1%, 95% CI − 0.5 to 16.7%. The RMST was 33.6 days in the CRRT group and 37.4 days in the IHD group (RMST difference 3.8 days, 95% CI 0.9 to 8.0).

### Weighted analyses

Groups were modestly imbalanced at baseline for hypertension, immunosuppressive drugs, SOFA scores, and creatinine levels. However, inverse probability weighting allowed to adequately balance the groups on all predefined confounders (Table 1 and Additional file 1: Figure S6). The weighted Kaplan–Meier death rate at day 60 was 54.4% in the CRRT group and 46.5% in the IHD group (Fig. 2B) (HR 1.26, 95% CI 1.01 to 1.60). The weighted ARD was 7.9%, 95% CI − 0.7 to 16.1%). The weighted RMST was 33.9 days in the CRRT group and 37.4 days in the IHD group (weighted RMST difference 3.5 days, 95% CI 0.9 to 8.0).

In further IPTW analyses, ICU and hospital mortality did not differ between groups. Same holds true for survival with no RRT dependence at day 28, kidney function recovery within 28 days, RRT-free days at day 28, ICU-free days at day 28, and length of ICU stay. However, the number of ventilator-free days and vasopressor-free days at day 28 were significantly higher in the IHD group than in CRRT group (Table 2).

**Table 2:** Average effect of CRRT versus IHD on secondary outcomes in the original and inverse probability of treatment weighted samples

Outcome	CRRT group (*n* = 269)	IHD group (*n* = 274)	RR or Difference	(95% CI)	Weighted RR or weighted difference	(95% CI)
Hospital mortality	146 (54.2%)	132 (48.3%)	1.12	(0.95 to 1.32)	1.13	(0.94 to 1.34)
ICU mortality	130 (48.3%)	114 (41.6%)	1.16	(0.96 to 1.40)	1.14	(0.93 to 1.39)
Survival with no RRT dependence at day 28	117 (43.5%)	132 (48.0%)	0.91	(0.75 to 1.10)	0.92	(0.75 to 1.13)
Kidney recovery at day 28	123 (45.7%)	134 (48.9%)	0.93	(0.78 to 1.12)	1.00	(0.83 to 1.21)
RRT-free days at day 28	11.5 (12.0)	13.4 (12.6)	− 1.9	(− 3.9 to 0.2)	− 1.6	(− 3.9 to 0.4)
Ventilator-free days at day 28	8.0 (10.0)	9.9 (10.8)	− 1.9	(− 3.7 to − 0.2)	− 1.9	(− 3.4 to − 0.1)
Vasopressor-free days at day 28	11.6 (11.7)	14.1 (12.3)	− 2.5	(− 4.5 to − 0.5)	− 2.1	(− 4.1 to − 0.1)
ICU-free days at day 28	6.0 (8.6)	7.7 (9.5)	− 1.6	(− 3.2 to − 0.1)	− 1.4	(− 3.1 to 0.2)
Length of ICU stay, days	14.2 (15.4)	14.4 (20.8)	− 0.2	(− 3.8 to 2.5)	0.1	(− 3.0 to 2.6)

Data are mean (SD) or *n* (%). All results are pooled over the imputed datasets. *RRT* renal replacement therapy, *CRRT* continuous renal replacement therapy, *IHD* intermittent hemodialysis, *ICU* intensive care unit, *RR* risk ratio, *CI* confidence interval

The overlap weighting (OW) analysis of the primary endpoint is presented in the supplementary appendix (Additional file 1: Figure S1). This sensitivity analysis did not change the magnitude of the treatment effect.

### Complementary analysis

**Fig. 3:**
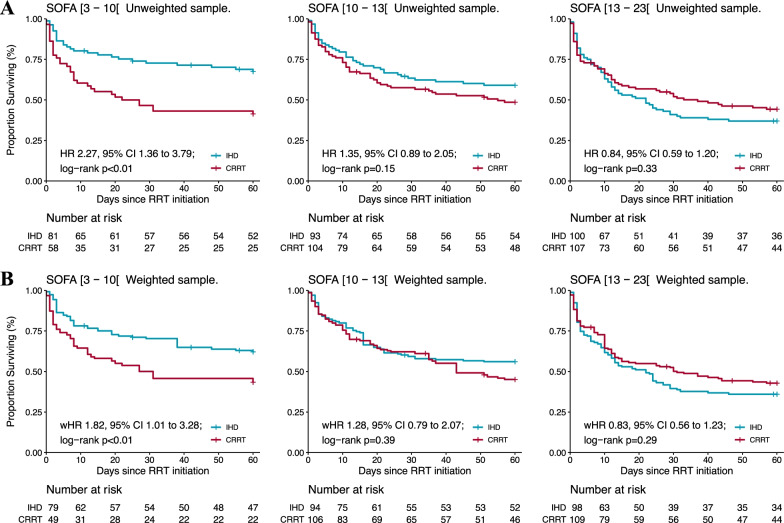
Treatment effect heterogeneity assessment: Probability of survival in the unweighted samples (**A**) and in the IPTW samples (**B**) stratified by thirds of baseline risk (SOFA score). *HR* hazard ratio, *IHD* intermittent hemodialysis, *CRRT* continuous renal replacement therapy

In an analysis stratified by thirds of SOFA scores, we found that IHD was associated with greater 60-day survival as compared to CRRT in less severely ill patients (weighted HR 1.82, 95% CI 1·01 to 3·28; *p* < 0.01 for patients with SOFA 3–10). In contrast, we found no evidence of a survival difference in more severely ill patients (Fig. 3).

## Discussion

This study compared continuous and intermittent RRT for the first session in critically ill patients with severe AKI. Continuous renal replacement therapy was associated with shorter survival within 60 days and with longer duration of mechanical ventilation and vasopressor support than IHD. This association persisted after adjustment for confounders through propensity scores methods. Moreover, in a complementary analysis, we showed that this difference was mainly the result of the poorer outcome of the less severely ill patients allocated to CRRT as compared to those allocated to IHD. These results may inform the debate on RRT modalities in critically ill patients.

The present COVID crisis is associated with a shortage of RRT devices [7], and innovative solutions are needed to solve this problem [8]. Intermittent hemodialysis allows faster removal of uremic toxins and control of electrolyte and acid–base disturbance than CRRT. This makes the same IHD machine available for several patients in a same day, particularly in countries where IHD is provided by usual ICU nurses, as is the case in France. In these conditions, it is of utmost importance to reassess the actual safety of IHD in critically ill patients. We took advantage of the fact that both studies included in the present analysis mandated very precise settings in order to maximize tolerance of IHD [9, 13, 15, 26]. In the present study, the choice of modality of RRT did not affect survival outcomes in the most severely ill patients. This result contradicts some common opinion [3] and guidelines [17] that promote CRRT for these patients. An explanation for this finding may be that any effect of RRT modality on prognosis was likely to be obscured by the severity of illness. In contrast, the less severely ill patients (according to the SOFA score) benefited significantly (*p* < 0.01) from IHD. This benefit of IHD in these patients may be explained by the short duration of RRT sessions which permits patients to be mobilized for nursing, early mobilization and medical procedure (such as CT scan or operating theater).

The KDIGO guidelines give a weak recommendation in favor of CRRT [17]. In contrast, French recommendations stipulate the equivalence of the two techniques [16]. Large multicenter randomized studies [12, 27] and a meta-analysis of the Cochrane Collaboration involving 15 RCTs and a total of 1550 patients concluded that CRRT did not differ from IHD with respect to mortality and kidney recovery [28]. Worthy of note is the fact that one randomized study [29] reported a higher mortality with CRRT than with IHD. Moreover, an individual patient data meta-analysis reported that time to cessation of RRT through 28 days was longer when CRRT was used as the initial modality of RRT [30]. Despite this, some key opinion leaders continue to favor CRRT over IHD in particular because of the alleged although unproven superiority of the former in hemodynamically unstable patients. This contention is however not supported by the conclusion of the Cochrane meta-analysis [28] which showed no difference in the occurrence of hemodynamic instability or hypotension or need for escalation of vasopressor therapy between RRT modalities. Then, there is no evidence-based data to support the fear of IHD in critically ill patients. Worthy of note is the fact that studies included in the Cochrane meta-analysis were published more than ten years ago whereas the prognosis of critically ill patients markedly improved in recent years [10, 11] and RRT modalities and indications were refined. For instance, mortality rate was more than 65% in both arms of the largest multicenter RCT comparing CRRT and IHD that was published in 2006 [12]. In contrast, recent RCTs on RRT indication (in which both methods were used) found that mortality was comprised between 43 and 58% [13–15].

This simple finding would justify a reappraisal of the respective merits and dangers of RRT techniques and the performance of new RCT comparing RRT modalities in a large cohort of patients with pre-specified RRT indications. This is all the most reasonable as the present study shows that compared to CRRT, mortality was not increased in a large population of critically ill patients when IHD was used as the first RRT session. The first session is frequently hampered by hemodynamic instability since patients may not be stabilized at the time of initiation of the technique. There is no reason to believe that subsequent RRT sessions would be less tolerated than the first one. In such condition, focus on the first modality initiated should have favored CRRT. Finding an association of opposite direction in fact strengthens our conclusions.

Our study included 543 critically ill patients (a number superior to the largest RCT included in the meta-analysis of the Cochrane [28]) who received RRT within twelve hours after the occurrence of severe AKI. The use of strict criteria for RRT initiation and the substantial overlap of the propensity score distribution (see Additional file 1: Figure S3) led to mimic the intention to treat analysis from an RCT. Indeed, the moderate imbalance of baseline characteristics was adequately corrected by inverse probability weighting (all SMDs less than 10%).

Our study has limitations. First, even though the present study used high-quality data from two large RCTs, it was not a RCT itself and bias could have arisen because of unknown or unmeasured confounders. However, after we applied robust methods to draw inferences from observational data, all the known and clinically relevant prognostic variables were balanced between groups (see Additional file 1: Figure S3 and S6). Second, we only analyzed patients allocated to the early group of AKIKI and IDEAL-ICU studies. However, time from ICU admission to RRT initiation was short and similar to those in previous RCTs, as well as reason for initiation [12].

The advantage of considering the early arm only stems from the fact that RRT was started just after randomization occurred (2–3 h). Then, baseline characteristics were unlikely to change noticeably. This would not be the case if we also included the delayed arm where RRT was initiated 48 to 52 h later than in the early arm. In such conditions, it would not be possible to eliminate major confounding. Third, patients receiving CRRT had higher cardiovascular and total-SOFA scores at baseline. Even if we used high-quality methodology with a propensity score analysis, confounding factors may persist. For instance, fluid overload which is difficult to accurately assess may have played a role.

Forth, even though the HR and RMSTD pointed to large treatment effect, these results may be fragile as statistical significance may depend on only a few events. However, a recent systematic review on critical care trials showed that for the trials with a statistically significant mortality difference, the fragility index was 4 (IQR 1–20) [31]. Fifth, when we considered 60-days survival through ARD, as opposed to HR and RMSTD, the significance of the association between CRRT and higher mortality was lost. Yet, this may just be a consequence of a lack of power in the ARD analyses: ARD assesses survival at a particular time point, whereas RMSTD and HR take advantage of all the information accrued from baseline through day 60. Sixth, we did not assess immediate adverse event such as hypotension, arrhythmia and increase in vasopressor during RRT session. Seventh, we did not assess a potential advantage on fluid control of CRRT over IHD. However, patient-centered outcomes such as duration of ICU and hospital stay may be affected by fluid balance but did not differ according to RRT modality. Eighth, a significant proportion of patients (almost 20%) were switched from a modality to another. However, the objective of the study was to assess the impact of the first RRT modality on prognosis and this first RRT modality was continued for a long duration in both groups (almost 6 days).

It should be noted that the clinical decision for choosing the optimal RRT mode is not only relying on outcome data but also on other factors such as practicability, availability of machines and the existence of trained nurse for IHD, which is the case in French ICUs.

## Conclusion

In this secondary analysis of two large multicenter randomized controlled trials on RRT timing, CRRT as first modality did not provide any benefit in term of survival within 60 days or kidney recovery and might be associated with less favorable outcome in patients with lesser severity of disease. Our results should merely be viewed as generating an unexpected hypothesis and an impetus for performing new large RCTs comparing RRT modalities.

## Supplementary Information

**Additional file 1.** **Table S1.** Balance of prognostic score and centre effects before and after inverse probability of treatment weighting. **Table S2.** Baseline characteristics of patients in the two groups after overlap weighting. **Figure S1.** Probability of survival in the overlap weighted sample. **Figure S2.** Bubble plot evaluating the association between centre-specific random effect for treatment allocation and centre-specific random effect for prognosis. **Figure S3.** Propensity score distribution in the original (A) and inverse probability of treatment weighted (B) samples. **Figure S4.** Propensity score (including centres as random effects) distribution in the original (A) and overlap weighted (B) samples. **Figure S5.** Fraction of missing data for each variable in the original sample. **Figure S6.** Standardised mean difference for each potential confounder in the unweighted, IPTW and OW samples.

## Data Availability

The datasets used during the current study are available from the corresponding author on reasonable request.

## Abbreviations

RRT: Renal replacement therapy
AKI: Acute kidney injury
IHD: Intermittent hemodialysis
CRRT: Continuous renal replacement therapy
RCT: Randomized controlled trial
ICU: Intensive care unit
AKIKI: Artificial Kidney Initiation in Kidney Injury
IDEAL-ICU: Initiation of dialysis early versus delayed in intensive care unit
KDIGO: Kidney Disease Improving Global Outcomes
SOFA: Sepsis‐related Organ Failure Assessment
HR: Hazard ratio
IPTW: Inverse probability of treatment weighting
SMD: Standardized mean differences
RR: Risk ratio
ARD: Absolute risk difference
MD: Mean difference (MD)
RMST: Restricted mean survival time
