# Supplementary material for: Continuous renal replacement therapy versus intermittent hemodialysis as first modality for renal replacement therapy in severe acute kidney injury: a secondary analysis of AKIKI and IDEAL-ICU studies
Source: Crit Care. 2022 Apr 4;26:93. doi: 10.1186/s13054-022-03955-9 (PMC8981658; doi:10.1186/s13054-022-03955-9)
Supplement: Supplementary file 1 — Additional file 1. Table S1. Balance of prognostic score and centre effects before and after inverse probability of treatment weighting. Table S2. Baseline characteristics of patients in the two groups after overlap weighting. Figure S1. Probability of survival in the overlap weighted sample. Figure S2. Bubble plot evaluating the association between centre-specific random effect for treatment allocation and centre-specific random effect for prognosis. Figure S3. Propensity score distribution in the original (A) and inverse probability of treatment weighted (B) samples. Figure S4. Propensity score (including centres as random effects) distribution in the original (A) and overlap weighted (B) samples. Figure S5. Fraction of missing data for each variable in the original sample. Figure S6. Standardised mean difference for each potential confounder in the unweighted, IPTW and OW samples. [file 13054_2022_3955_MOESM1_ESM.docx]

**Supplementary Appendix**

**Continuous renal replacement therapy versus intermittent haemodialysis as first modality for renal replacement therapy in severe acute kidney injury in intensive care unit**

**Table of Contents**

[Tables 2](#_Toc66285207)

[Table S1. Balance of prognostic score and centre effects before and after inverse probability of treatment weighting. 2](#_Toc66285208)

[Table S2. Baseline characteristics of patients in the two groups after overlap weighting. 3](#_Toc66285209)

[Figures 4](#_Toc66285210)

[Figure S1. Probability of survival in the overlap weighted sample. 4](#_Toc66285211)

[Figure S2. Bubble plot evaluating the association between centre-specific random effect for treatment allocation and centre-specific random effect for prognosis. 5](#_Toc66285212)

[Figure S3. Propensity score distribution in the original (A) and inverse probability of treatment weighted (B) samples. 6](#_Toc66285213)

[Figure S4. Propensity score (including centres as random effects) distribution in the original (A) and overlap weighted (B) samples. 6](#_Toc66285214)

[Figure S5. Fraction of missing data for each variable in the original sample. 7](#_Toc66285215)

[Figure S6. Standardised mean difference for each potential confounder in the unweighted, IPTW and OW samples. 8](#_Toc66285216)

# Tables

## Table S1. Balance of prognostic score and centre effects before and after inverse probability of treatment weighting.

Mixed effect Cox proportional hazard regression was used to derive a multivariable prediction model for 60-days survival (primary outcome). Centres were used as the covariate for random effects while all confounding variables (see main text) were used for fixed effects. We then allocated each patient their prognostic score and corresponding centre-specific random effects (best linear unbiased prediction, BLUP). In the unweighted and inverse probability of treatment weighting (IPTW) pseudo populations, we assessed the balance of the prognostic score and centre effects between the two groups.

|  | **CRRT group** | **IHD group** | **SMD (%)** | **P value** |
| --- | --- | --- | --- | --- |
| Unweighted sample |  |  |  |  |
| Number of patients | 269 | 274 |  |  |
| Prognostic score, mean (SD) | 2.71 (0.59) | 2.68 (0.62) | 4.8 | 0.56 |
| Centre effects, mean (SD) | 0.02 (0.14) | 0.01 (0.14) | 5.2 | 0.41 |
| IPTW sample |  |  |  |  |
| Number of patients (sum of weights) | 268.2 | 271.9 |  |  |
| Prognostic score, mean (SD) | 2.69 (0.59) | 2.70 (0.61) | 1.1 | 0.91 |
| Centre effects, mean (SD) | 0.01 (0.14) | 0.01 (0.14) | 0.1 | 0.99 |

CCRT=Continuous Renal Replacement Therapy.

IHD=Intermittent Haemodialysis.

SMD=Standardised Mean Difference, expressed as a percentage.

IPTW=Inverse Probability of Treatment Weighting.

## Table S2. Baseline characteristics of patients in the two groups after overlap weighting.

Numbers of patients reported are the sum of stabilised weights.

| **Characteristic** | **CRRT group (n=69.4)** | **IHD group (n=70.2)** | **SMD (%)** | **P value** |
| --- | --- | --- | --- | --- |
| Age, years | 66.9 (12.9) | 66.8 (12.9) | 0.5 | 0.96 |
| Female sex | 23.9 (34.5%) | 24.3 (34.6%) | 0.3 | 0.98 |
| Serum creatinine, μmol/L | 86.7 (29.0) | 86.3 (28.8) | 1.5 | 0.89 |
| Coexisting conditions |  |  |  |  |
| Chronic kidney disease | 6.5 (9.3%) | 7.7 (11.0%) | 5.3 | 0.66 |
| Hypertension | 38.2 (55.0%) | 38.2 (54.4%) | 1.1 | 0.92 |
| Diabetes mellitus | 15.1 (21.7%) | 15.2 (21.6%) | 0.1 | 0.99 |
| Congestive heart failure | 6.3 (9.1%) | 6.4 (9.1%) | <0.001 | >0.99 |
| Cirrhosis | 7.5 (10.8%) | 7.7 (11.0%) | 0.5 | 0.96 |
| Respiratory disease | 8.2 (11.9%) | 8.2 (11.6%) | 0.7 | 0.95 |
| Cancer | 11.5 (16.6%) | 11.7 (16.6%) | 0.2 | 0.99 |
| AIDS | 0·8 (1·1%) | 0.8 (1.1%) | 0.3 | 0.98 |
| Immunosuppressive drugs | 3.0 (4.3%) | 3.2 (4.6%) | 1.5 | 0.89 |
| Organ transplantation | 0.4 (0.6%) | 0.4 (0.6%) | 0.1 | >0.99 |
| SOFA score at inclusion (0 to 24) | 11.5 (3.2) | 11.5 (3.1) | 2.1 | 0.85 |
| Renal SOFA (1 to 5) | 3.7 (1.1) | 3.7 (1.1) | 2.4 | 0.83 |
| Haemodynamic SOFA (1 to 5) | 4.4 (1.3) | 4.4 (1.3) | 2.1 | 0.86 |
| Liver SOFA (1 to 5) | 1.8 (1.1) | 1.8 (1.1) | 4.0 | 0.73 |
| Neurologic SOFA (1 to 5) | 2.4 (1.6) | 2.4 (1·5) | 0.5 | 0.96 |
| Coagulation SOFA (1 to 5) | 3.2 (1.7) | 3.1 (1.6) | 2.9 | 0.80 |
| Body weight, kg | 86.7 (29.0) | 86.3 (28.8) | 1.5 | 0.89 |
| Laboratory values |  |  |  |  |
| Serum creatinine, μmol/L | 282.0 (124.4) | 289.4 (119.3) | 6.1 | 0.59 |
| Serum urea, mmol/L | 19.9 (9.4) | 19.7 (8.9) | 1.7 | 0.88 |
| Serum potassium, mmol/L | 4.41 (0.78) | 4.41 (0.77) | 0.1 | 0.99 |
| Arterial blood pH | 7.30 (0.09) | 7.30 (0.09) | <0.1 | >0.99 |

Data are n (%) or mean (SD).

SMD=Standardised Mean Difference, expressed as a percentage.

SOFA score=Sequential Organ Failure Assessment score.

# Figures

## Figure S1. Probability of survival in the overlap weighted sample.

The weighted Kaplan-Meier death rate at day 60 was 57·1% in the CRRT group and 49·5% in the IHD group (overlap weighted ARD 7·6%, 95% CI –2·4% to 18·0%).

wHR=weighted Hazard Ratio.

wRMSTD=weighted Restricted Mean Survival Time.

CCRT=Continuous Renal Replacement Therapy.

IHD=Intermittent Haemodialysis.

## Figure S2. Bubble plot evaluating the association between centre-specific random effect for treatment allocation and centre-specific random effect for prognosis.

Each centre was allocated its centre-specific random effect on treatment allocation and its centre-specific random effect on prognosis from the propensity score and prognostic score models respectively. The propensity score model was fitted through mixed-effects logistic regression with all confounding variables as fixed effects (see main text) and a random centre effect. The prognostic model predicted 60-days survival and was fitted through mixed effect Cox proportional hazard regression with treatment allocation and the same confounding variables as fixed effects (see main text) as well as a random centre effect. We found no associational pattern between centre-specific random effect for treatment allocation and centre-specific random effect for prognosis, thus suggesting that centres were not confounding variables and omission of centres from the propensity score model did not result in a violation of the ignorability assumption.

## Figure S3. Propensity score distribution in the original (A) and inverse probability of treatment weighted (B) samples.

The overlap of the distributions allows to assess the positivity assumption.

**** ****

## Figure S4. Propensity score (including centres as random effects) distribution in the original (A) and overlap weighted (B) samples.

The overlap of the distributions allows to assess the positivity assumption.

CCRT=Continuous Renal Replacement Therapy.

IHD=Intermittent Haemodialysis.

## Figure S5. Fraction of missing data for each variable in the original sample.

RRT=Renal Replacement Therapy.

## Figure S6. Standardised mean difference for each potential confounder in the unweighted, IPTW and OW samples.

IPTW=Inverse Probability of Treatment Weighting.

OW=Overlap Weighting.

SMD=Standardised Mean Difference.
